# Supplementary material for: Comprehensive profiling reveals Sialyl-Tn upregulation and prognostic value in prostate cancer
Source: bioRxiv. 2026 Apr 15:2026.04.14.718221. Preprint. [Version 1] doi: 10.64898/2026.04.14.718221 (PMC13105074; doi:10.64898/2026.04.14.718221)

bioRxiv preprint doi: https://doi.org/10.64898/2026.04.14.718221; this version posted April 15, 2026. The copyright holder for this preprint (which was not certified by peer review) is the author/funder, who has granted bioRxiv a license to display the preprint in perpetuity. It is made available under aCC-BY 4.0 International license.

Supplementary Figure 1. **Expression of sTn does not correlate with prostate cancer Gleason grade**. Analysis of sTn levels in TMA cohort 1 and 2 and correlation with Gleason grade. (**A**) Analysis of sTn levels in prostate cancer tissue samples from TMA cohort 1 shows there is no significant difference in sTn levels in Gleason grade 1-2 tumours compared to Gleason grade 3-5 tumours (n=79, unpaired t test, p=0.2820). (**B**) Analysis of sTn levels in 90 prostate cancer tissue samples from TMA cohort 2 shows there is no significant difference in sTn levels in Gleason grade 1-2 tumours compared to Gleason grade 3-5 tumours (n=90, unpaired t test, p=0.8917).

Supplementary Figure 1

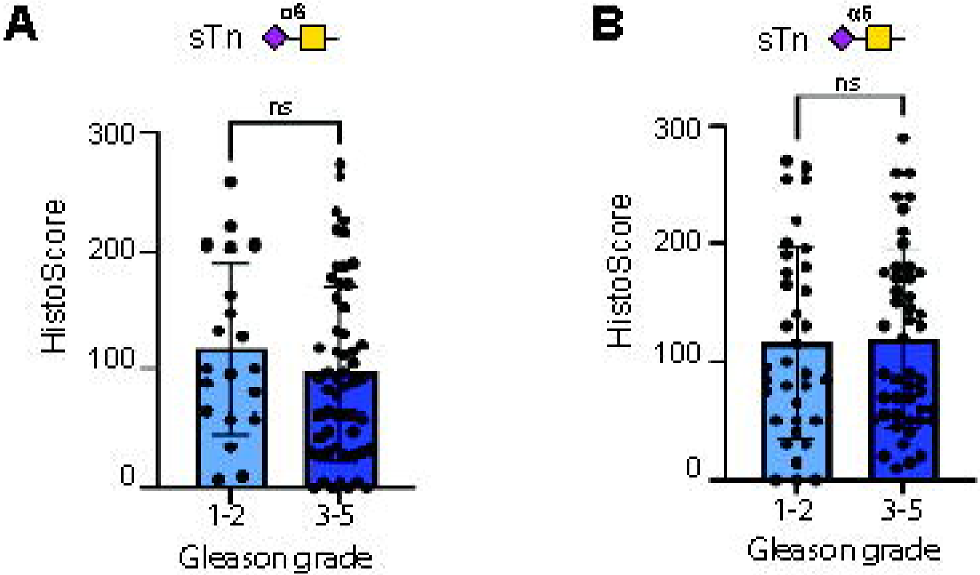

Supplement: 1 [file NIHPP2026.04.14.718221v1-supplement-1.pdf]
